# Quality of life assessment in patients undergoing partial and total thyroidectomy

**DOI:** 10.1590/0100-6991e-20253870-en

**Published:** 2025-11-24

**Authors:** PEDRO HENRIQUE SERRA CARVALHO DOS SANTOS, ROBERTA ZAMBO GALAFASSI, GIULIA FERNANDES MANHÃES RODRIGUES LOPES, LEIZIANE ASSUNÇÃO ALVES GUIMARÃES, FATIMA ROSALIA HARTELSBERGER BOBADILLA, INÊS NOBUKO NISHIMOTO, ROGÉRIO APARECIDO DEDIVITIS

**Affiliations:** 1 - Faculdade de Ciências Médicas de Santos - Centro Universitário Lusíada - Santos - SP - Brasil; 2 - Hospital Ana Costa - Santos - SP - Brasil; 3 - Centro Universitário Lusíada - Santos - SP - Brasil; 4 - Faculdade de Medicina da Universidade de São Paulo, Cirurgia de Cabeça e Pescoço - São Paulo - SP - Brasil

**Keywords:** Thyroidectomy, Quality of Life, Postoperative Complications, Dysphonia, Hypoparathyroidism, Tireoidectomia, Qualidade de Vida, Complicações Pós-Operatórias, Disfonia, Hipoparatireoidismo

## Abstract

**Introduction:**

Thyroidectomy, which consists of partial or total removal of the thyroid gland, is a commonly performed surgery to treat various thyroid diseases. In recent years, the trend has been toward partial thyroidectomies, due to their association with lower complication rates and the fact that they may be sufficient for adequate management.

**Objective:**

To evaluate the impact of partial and total thyroidectomy on quality of life.

**Methods:**

This study used the ThyPro questionnaire, which assesses the quality of life of patients with thyroid disorders, to investigate the impact of different thyroidectomy approaches on patients’ quality of life.

**Results:**

The postoperative complications were transient unilateral recurrent nerve paralysis (6.5%) and transitory hypoparathyroidism (22.4%). The quality of life outcomes after thyroidectomy were favorable. The extension of the thyroidectomy did not present statistical difference (p = 0.982). Significantly lower scores were associated with female gender and neck dissection.

**Conclusion:**

Quality of life scores were high, with lower scores among women and neck dissection.

## RESUMO

**Objetivo:**

A tireoidectomia, remoção parcial ou total da glândula tireoide, é uma cirurgia comumente realizada para diversas doenças da tireoide. Recentemente, tem-se observado uma tendência pelo uso de tireoidectomias parciais, devido às taxas de complicações mais baixas e resultados terapêuticos adequados.

**Objetivo:**

Avaliar o impacto da tireoidectomia parcial e total na qualidade de vida.

**Métodos:**

O questionário ThyPRO-39 foi utilizado para avaliar os resultados específicos de qualidade de vida relacionados à tireoide em pacientes que se submeteram a diferentes abordagens de tireoidectomia.

**Resultados:**

As complicações pós-operatórias foram paralisia unilateral transitória do nervo recorrente (6,5%) e hipoparatireoidismo transitório (22,4%). Os resultados de qualidade de vida após a tireoidectomia foram favoráveis. A extensão da tireoidectomia não apresentou diferença estatística (p = 0,982). Escores significativamente mais baixos foram associados ao gênero feminino e ao esvaziamento cervical.

**Conclusão:**

Os escores de qualidade de vida foram elevados, com escores mais baixos entre as mulheres e na presença de esvaziamento cervical.

## INTRODUCTION

Thyroidectomy can be partial or total^1^. With the increase in safety and efficacy of these procedures, there is a growing preference for less than total thyroidectomy, as it is associated with reduced complication rates while providing adequate management for low-risk tumors and benign conditions^2^ ^,^ ^3^. 

Thyroid surgery complications may impact patients’ quality of life. The main ones are permanent or transient hypocalcemia, permanent or transient recurrent laryngeal nerve paralysis,^5^ as well as hemorrhage and hematoma. Total thyroidectomy is associated with higher hypoparathyroidism rates compared with hemithyroidectomy^4^ ^,^ ^5^.

In recent years, the discussion about the quality of life in patients undergoing thyroidectomy has gained prominence. Health-related quality of life can be defined as the global impact that diseases and their treatments have on all relevant dimensions of the patient’s life. This concept can only be evaluated by the patient himself/herself, as it is subjective and includes different aspects of well-being, such as physical, mental, and social. Standardized questionnaires are used to measure it^6^.

An instrument that is used to evaluate specific thyroid treatment results is the Thyroid-Related Patient-Reported Outcome (ThyPRO) questionnaire, developed by Watt et al.^7^ ^,^ ^8^ at the Rigshospitalet, in Copenhagen, Denmark, which evaluates the quality of life of patients with benign thyroid disorders, including thyroid dysfunction and goiter. The questionnaire includes 12 domains covering physical and mental aspects, symptoms, well-being, function, as well as the impact of thyroid diseases on social and daily life and, in general, on the quality of life of patients with benign thyroid disorders. The PRO suffix denotes “patient-reported outcome”. The questionnaire originally underwent the four phases of development: phase I (generation edition), where questions related to quality of life and possible relevance were identified in relation to thyroid diseases; phase II (operationalization), where selected relevant questions were converted to items of a draft questionnaire; phase III (pre-test), where a draft of the questionnaire was tested and revised based on the cognitive interview technique; and phase IV (quantitative validation), where the internal consistency, reliability, and validity (Cronbach’s) of the constructed scales were tested in a large sample of patients. The questionnaire has been validated and considered useful in clinical studies^9^ ^,^ ^10^.

In 2015, an abbreviated version of this questionnaire, with 39 questions, was developed and named ThyPRO-39. This new questionnaire showed good measurement properties and was recommended for clinical use^11^. This same version was translated and satisfactorily validated into Brazilian Portuguese and is known as ThyPRO-39br^12^.

Although initially constructed to evaluate the results in benign thyroid diseases, ThyPRO can be applied to patients undergoing thyroidectomy, regardless of surgical indication and including suspected or confirmed malignancy^13^ ^,^ ^14^. Based on this, in our study we did not consider malignancy as an exclusion factor.

The aim of this study was to investigate the differences in the impact on the patient’s quality of life by comparing total and partial thyroidectomy and to analyze potential risk factors for worse quality of life in patients undergoing the procedure due to benign and malignant disease.

## METHODS

The research was approved by the Human Ethics in Research Committee (CEPSH) of the Lusíadas University Center (UNILUS), under protocol number 895/2024, CAAE number 80501324.9.0000.5436.

This is a cross-sectional, observational study, in which we applied a questionnaire on quality of life in patients with thyroid diseases, the Thyroid-Related Patient-Reported Outcome-39 (ThyPRO-39), validated for Brazilian Portuguese by Dr. Jônatas Catunda^12^.

We included consecutive patients undergoing thyroidectomy and being followed up at the Head and Neck Surgery Center of Santos. They underwent total or partial thyroidectomy between 2021 and 2023, with a postoperative follow-up time of at least twelve months. Considering previous validation^13^ ^,^ ^14^, we enrolled patients operated on for benign and malignant diseases. We excluded those under 18 years of age or unable to perform any of the study assessments. We collected epidemiological and clinical data from medical records. 

Patients were asked to fill out the ThyPRO-39br^12^ on the Google Forms platform. The first field to be filled in was the Informed Consent Form and then the questionnaire. Data collection was carried out between May and September 2024.

After answering the questions for sample segmentation, the participant answered the ThyPRO-39br questionnaire. Responses are scored from 0 to 4, following a Likert scale (where ‘’0’’ equals ‘’no’’ and ‘’4’’ equals ‘’very’’), always considering the perception during the last four weeks. 

The results are distributed in 13 aspects or scales, and a final composite result generates a score from 0 to 100 for each of them. The higher the score, the higher the frequency of symptoms of the respective scale and the greater the impact on quality of life. 

The scales are: 1. Goiter symptoms; 2. Hyperthyroidism symptoms; 3. Hypothyroidism symptoms; 4. Ocular symptoms; 5. Fatigue; 6. Cognitive problems; 7. Anxiety; 8. Depression; 9. Emotional; 10. Impact on social life; 11. Impact on daily life; 12. Complaints of appearance; and 13. Quality of life in general. The way the results of the ThyPRO-39br are calculated are in the Annex.

The outcome of the methods used was to create comparative scores of the results of the ThyPRO-39br scales with the categorical variables sex, age, anatomopathological outcome, presence of thyroiditis, thyroidectomy extension, neck dissection, radioiodine therapy, unilateral nerve palsy, loss of vocal extension, hypoparathyroidism, need for definitive calcium replacement, and need for definitive thyroid hormone replacement.

As for statistical analysis, we used the frequency distribution to describe the categorical variables (number of cases and relative percentage) and the measures of central tendency (mean and median) and variability (minimum, maximum and standard deviation) for the numerical variables. The associations between the scores and independent categorical variables with two categories were assessed with the Mann-Whitney U test, and with three or more categories, with the Kruskal-Wallis test. We adopted a significance level of 5% for all statistical tests. We used the statistical software Stata, version 18, to perform all statistical analyses^15^ .

## RESULTS

We included 107 patients who answered the questionnaire. There was a predominance of females (77.6%) and the mean age was 50.9 years. Regarding the anatomopathological diagnosis, 57.9% were goiter or adenoma, and thyroiditis was not observed in 83.2%. Total thyroidectomy was performed in 81.3% (87); 86.9% did not undergo neck dissection; and 92.5% did not receive adjuvant radioiodine therapy. Regarding complications, seven patients (6.5%) had transient unilateral recurrent nerve palsy, 24 (22.4%) had hypoparathyroidism, and 98.1% (105) did not require calcium replacement (Table 1).

**Table 1:** Sample demographics and clinical variables - 107 patients.

Variable	Category / Measurements	Freq. (%) / Measures
Sex	Female Male	83 (77.6) 24 (22.4)
Age (years)	Range Median Mean (Standard Deviation)	25 - 84 51 50.9 (13.4)
Anatomopathological	Goiter/Adenoma Well-differentiated carcinoma	62 (57.9) 45 (42.1)
Thyroiditis	No Yes	89 (83.2) 18 (16.8)
Extension of thyroidectomy	Partial Total	20 (18.7) 87 (81.3)
Neck dissection	No Single-sided central compartment Bilateral central compartment Lateral Modified radical	93 (86.9) 1 (0.9) 0 12 (11.2) 1 (0.9)
Neck dissection	No Yes	93 (86.9) 14 (13.1)
Radioiodine therapy	No Yes	99 (92.5) 89 (7.5)
Unilateral nerve palsy	No Transitory Definitive	100 (93.5) 7 (6.5) 0
Hypoparathyroidism	No Yes	83 (77.6) 24 (22.4)
Calcium replacement	No Yes	105 (98.1) 2 (1.9)
Thyroid hormone replacement	No Hormone replacement Suppression of TSH	22 (20.6) 47 (43.9) 38 (35.5)

The ThyPRO-39 questionnaire allowed the sample to be distributed according to the symptoms scales. Among the symptoms reported, those standing out were fatigue, with a median of 33 and an average score of 42.2, depression, with a median of 22 and a mean of 28.1, and anxiety, with a median of 18 and a mean of 23.6. The emotional aspect showed a median of 28 and a mean of 32.8. Cognitive problems had a median of 14 and a mean of 20.2 (Table 2).

**Table 2:** Sample distribution according to symptom scales - 107 cases.

Variable	Measures	Measures
Goiter symptoms	Range Median Mean (standard deviation)	2 - 73 10 12.2 (13.8)
Hyperthyroidism symptoms	Range Median Mean (standard deviation)	2 - 84 8 11.6 (12.8)
Hypothyroidism symptoms	Range Median Mean (standard deviation)	0 - 87.5 6.25 15.8 (18.5)
Ocular symptoms	Range Median Mean (standard deviation)	1 - 68 1 8.3 (12.2)
Fatigue	Range Median Mean (standard deviation)	0 - 100 33 42.2 (28.7)
Cognitive problems	Range Median Mean (standard deviation)	1 - 76 14 20.2 (20.5)
Anxiety	Range Median Mean (standard deviation)	1 - 79 18 23.6 (20.5)
Depression	Range Median Mean (standard deviation)	0 - 97 22 28.1 (22.4)
Emotional	Range Median Mean (standard deviation)	1 - 95 28 32.8 (25.2)
Variable	Measures	Measures
Impact on social life	Range Median Mean (standard deviation)	0 - 75 0 11.9 (17.8)
Impact on day-to-day life	Range Median Mean (standard deviation)	0 - 98 0 17.2 (22.8)
Appearance complaints	Range Median Mean (standard deviation)	1 - 87 1 18.1 (23.1)

The average overall impact on quality of life was 18.9 (Table 3).

**Table 3:** Sample distribution according to the aspect Quality of Life in General and the composite symptoms scale - 107 cases.

Variable	Measures	Measures
Quality of life in general	Range Median Mean (standard deviation)	0 - 100 0 18.9 (26.6)
Composite symptoms scale	Range Median Mean (standard deviation)	1.1 - 71.6 23.9 25.8 (16.7)

There was a predominance of goiter symptoms in females and among individuals who required calcium replacement, and these symptoms were more pronounced among individuals who underwent neck dissection. Females and those under 50 years of age had greater hyperthyroidism symptoms. Patients undergoing total thyroidectomy had higher scores for hypothyroidism symptoms, while neck dissection showed an increase in the manifestation of ocular symptoms. Female patients and those undergoing neck dissection had higher scores in the fatigue aspect. Female sex and hypoparathyroidism showed higher scores for anxiety. The depression aspect was higher among women, patients aged up to 50 years, and among those who underwent radioiodine therapy. The evaluation of the scores of the emotional sensitivity aspect revealed higher rates among women, aged up to 50 years and in well-differentiated thyroid carcinoma. The analysis of the impact on social life and the impact on day-to-day life aspects revealed higher scores in females and in case of neck dissection. Investigation of the appearance complaints aspect revealed a greater impact after total thyroidectomy.

Demographics and clinical variables did not show significant differences when related to the Quality of Life in General aspect, except for neck dissection (Table 4). As for the ThyPRO-39 composite scale, female sex and neck dissection were related to higher scores (Table 5).

**Table 4:** Demographics and clinical variables according to ThyPro Overall impact on quality of life aspects - 107 patients.

Variable	Measures	N	Range	Median	Medium (±SD)	p-value
Sex	Female Male	83 24	0 - 100 0 - 75	0 0	21.4 (± 27.9) 10.4 (± 19.4)	0.081
Age (years)	≤ 50 > 50	53 54	0 - 100 0 - 75	0 0	22.6 (± 29.5) 15.3 (± 23.0)	0.193
Anatomopathology	Goiter/Adenoma Well-differentiated carcinoma	62 45	0 - 75 0 - 100	0 0	17.7 (± 24.1) 20.5 (± 29.8)	0.896
Thyroiditis	No Yes	89 18	0 - 100 0 - 75	0 25	17.4 (± 26.5) 26.4 (± 26.4)	0.097
Extension of thyroidectomy	Partial Total	20 87	0 - 75 0 - 100	0 0	17.5 (± 23.1) 19.2 (± 27.4)	0.982
Neck dissection	No Yes	93 14	0 - 100 0 - 100	0 25	16.9 (± 25.0) 32.1 (± 33.1)	0.058
Radioiodine therapy	No Yes	99 8	0 - 100 0 - 100	0 25	17.9 (± 26.0) 31.2 (± 32.0)	0.108
Unilateral nerve palsy	No Transitory	100 7	0 - 100 0 - 50	0 0	19.2 (± 27.0) 14.3 (± 19.7)	0.822
Variable	Measures	N	Range	Median	Medium (±SD)	p-value
Hypoparathyroidism	No Yes	83 24	0 - 100 0 - 75	0 0	19.9 (± 27.0) 15.6 (± 25.3)	0.468
Calcium replacement	No Yes	105 2	0 - 100 0	0 0	19.3 (± 26.7) 0	NA
Thyroid hormone replacement	No Hormone replacement Suppression of TSH	22 47 38	0 - 75 0 - 75 0 - 100	0 0 0	13.6 (± 21.4) 19.1 (± 25.1) 21.7 (± 30.8)	0.669*

p-value obtained by the Mann-Whitney U test; * p-value obtained by the Kruskal-Wallis test; NA = Not statistically evaluable.

**Table 5:** Demographics and clinical variables according to ThyPro Composite scale - 107 patients.

Variable	Measures	N	Range	Median	Medium (±SD)	p-value
Sex	Female Male	83 24	1.1 - 71.6 2.3 - 46.6	27.3 12.5	28.6 (± 17.2) 16.0 (± 10.6)	<0.001
Age group (years)	≤ 50 > 50	53 54	2.3 - 71.6 1.1 - 56.8	28.4 20.4	29.5 (± 18.5) 22.2 (± 14.0)	0.051
Anatomopathology	goiter/adenoma Well-differentiated carcinoma	62 45	1.1 - 71.6 2.3 - 69.3	20.4 25.0	23.3 (± 15.9) 29.2 (± 17.4)	0.086
Thyroiditis	No Yes	89 18	1.1 - 69.3 3.4 - 71.6	22.7 25	25.5 (± 16.3) 27.4 (± 19.0)	0.780
Extension of thyroidectomy	Partial Total	20 87	3.4 - 56.8 1.1 - 71.6	20.4 25	21.4 (± 13.6) 26.8 (± 17.3)	0.220
Neck dissection	No Yes	93 14	1.1 - 71.6 20.4 - 67.0	20.4 30.1	24.3 (± 16.4) 35.5 (± 16.1)	0.018
Radioiodine therapy	No Yes	99 8	1.1 - 71.6 11.4 - 67.0	22.7 34.1	25.0 (± 16.2) 36.1 (± 21.1)	0.118
Unilateral nerve palsy	No Transitory	100 7	1.1 - 71.6 4.5 - 36.4	23.9 9.1	26.4 (± 16.8) 17.4 (± 13.4)	0.144
Hypoparathyroidism	No Yes	83 24	1.1 - 71.6 6.8 - 59.1	22.7 31.8	24.6 (± 17.2) 30.0 (± 14.5)	0.055
Calcium replacement	No Yes	105 2	1.7 - 71.6 19.3 - 35.2	23.9 27.3	25.8 (± 16.9) 27.3 (± 11.2)	0.757
Thyroid hormone replacement	Hormone replacement Suppression of TSH	22 47 38	3.4 - 56.8 1.1 - 71.6 2.3 - 69.3	17.6 23.9 27.3	20.7 (± 13.6) 24.3 (± 15.2) 30.6 (± 19.2)	0.135*

p-value obtained by the Mann-Whitney U test; * p-value obtained by the Kruskal-Wallis test.

## DISCUSSION

The quality-of-life results obtained in our study were good. One hundred and seven patients participated in the study and the overall impact on quality of life of ThyPRO-39 had a mean of 18.9, while the median for the composite symptom scale was 23.9. Symptoms predominated in females, among patients on calcium replacement, and among those who underwent neck dissection. Women tend to have higher levels of anxiety related to the diagnosis of thyroid nodule or cancer, even in low-risk cases. This amplifies the negative perception of residual symptoms and limitations in the postoperative period^16^.

Impairment in quality of life is more frequent in a one-year follow-up after thyroidectomy. This may have been attributed to postoperative complications such as hypocalcemia, vocal cord paralysis, or hematoma during the one-year postoperative period^17^. 

Post-thyroidectomy quality of life can vary between patients and depends on several factors, such as the presence of complications, the need for hormone replacement therapy, and adaptation to postoperative changes. Complications are not uncommon^18^, so the advantages of partial thyroidectomy, including lower risk of recurrent laryngeal nerve injury and hypoparathyroidism, are important. In addition, there is a growing awareness that thyroid replacement therapy imperfections and postoperative scarring have effects on well-being^19^. 

We found an association between hypoparathyroidism and anxiety. Partial thyroidectomy minimizes the risk of hypoparathyroidism. By preserving part of the thyroid and therefore the parathyroid glands on the unmanipulated side, surgery reduces the likelihood of damage or removal of the parathyroids. However, even in partial thyroidectomies, there is still some risk of hypoparathyroidism. Postoperative follow-up is important to monitor parathyroid function and calcium levels^20^ ^,^ ^21^.

Dysphonia after thyroidectomy is relatively common and can occur due to trauma to the vocal folds, intubation, or inflammation of the healing process. As the patients were evaluated one year after surgery, definitive vocal fold paralysis is the outcome with the potential to impact vocal quality. In partial thyroidectomy, it may have a lower risk compared to total thyroidectomy, in which both sides are manipulated^22^. We did not find dysphonia in long-term evaluations.

Neck scarring is one of the potential factors that can affect quality of life^23^. Patients are usually satisfied with their scars in the short term^24^. However, there is little evidence on the long-term evolution of patients’ perceptions^25^. Although it tends to improve over time, cervical scarring after thyroidectomy can compromise quality of life, especially in women, by accentuating anxiety, aesthetic concern, and social impact. Nonetheless, in most patients the scar is discreet and aesthetically acceptable, with high satisfaction. We did not find significant aesthetic complaints, but this was significantly higher in the total thyroidectomy group. The literature does not share these findings, and there is no significant difference in scar scores between hemithyroidectomy and total thyroidectomy, which would be intuitive, since the scar in both conditions tends to be similar^26^.

Reasons for a worse prognosis after thyroidectomy include fear of recurrence, adjustment to normal TSH, and postoperative complications^27^ ^,^ ^28^. The fact that scores related to mental health and vitality, as well as limitations due to physical or emotional problems, did not deteriorate within the period examined, signals that thyroidectomy does not affect patients’ long-term quality of life. The diagnosis and treatment of cancer can lead to a series of changes in the mental/psychological capacity of the patient, in the social role he plays in society, in his personal life and, finally, in everything that involves appearance^29^.

The persistence of higher levels of depression in patients requiring multiple levothyroxine dosage adjustments to maintain euthyroidism compared with patients not requiring adjustments may be due to depressive symptoms requiring a longer period of euthyroidism to disappear after surgical treatment. This explains the higher rates of hypothyroidism symptoms among patients who underwent total thyroidectomy in our study. Remission of depressive symptoms after pharmacological treatment of thyroid disease has been widely demonstrated^19^. 

The extent of thyroidectomy in the initial surgical management of differentiated thyroid carcinoma has generated significant debate. There are clear and widely accepted indications for total thyroidectomy: overt bilateral disease, extrathyroidal extension, aggressive variants, and regional or distant metastases. In the absence of these characteristics, different indications arise as to extension. One of the arguments to support hemithyroidectomy is that it has less impact on patients’ quality of life^30^.

The present series reveals good quality of life in patients undergoing thyroidectomy related to the low rate of complications. The limitations of this study are its retrospective nature and the fact that the sample size is moderate, though sufficient for statistical analysis.

## CONCLUSION

Quality of life after thyroidectomy was good overall, being influenced by specific clinical and demographic variables. Females had worse scores in multiple domains (fatigue, anxiety, depression, emotional sensitivity, social impact, and appearance). Neck dissection was associated with greater global impairment of quality of life, with increased symptoms of goiter, fatigue, social and ocular impact. Total thyroidectomy was associated with higher scores for hypothyroidism symptoms and worse aesthetic perception. Hypoparathyroidism correlated with greater anxiety, although it rarely required continuous calcium replacement. Radioiodine therapy and age <50 years were associated with greater depression and symptoms of hyperthyroidism.
